# Feasibility of cardiopulmonary exercise testing and training using a robotics-assisted tilt table in dependent-ambulatory stroke patients

**DOI:** 10.1186/s12984-015-0078-5

**Published:** 2015-09-26

**Authors:** Jittima Saengsuwan, Celine Huber, Jonathan Schreiber, Corina Schuster-Amft, Tobias Nef, Kenneth J. Hunt

**Affiliations:** Institute for Rehabilitation and Performance Technology, Division of Mechanical Engineering, Department of Engineering and Information Technology, Bern University of Applied Sciences, Burgdorf, Switzerland; ARTORG Center for Biomedical Engineering Research, Gerontechnology and Rehabilitation Research Group, University of Bern, Bern, Switzerland; Research Department, Reha Rheinfelden, Rheinfelden, Switzerland; Department of Physical Medicine and Rehabilitation, Faculty of Medicine, Khon Kaen University, Khon Kaen, Thailand

**Keywords:** Cardiopulmonary fitness, Exercise testing, Exercise training, Peak oxygen uptake, Robotics, Robotics-assisted tilt table, Stroke

## Abstract

**Background:**

We evaluated the feasibility of an augmented robotics-assisted tilt table (RATT) for incremental cardiopulmonary exercise testing (CPET) and exercise training in dependent-ambulatory stroke patients.

**Methods:**

Stroke patients (Functional Ambulation Category ≤ 3) underwent familiarization, an incremental exercise test (IET) and a constant load test (CLT) on separate days. A RATT equipped with force sensors in the thigh cuffs, a work rate estimation algorithm and real-time visual feedback to guide the exercise work rate was used. Feasibility assessment considered technical feasibility, patient tolerability, and cardiopulmonary responsiveness.

**Results:**

Eight patients (4 female) aged 58.3 ± 9.2 years (mean ± SD) were recruited and all completed the study. For IETs, peak oxygen uptake (V'O_2peak_), peak heart rate (HR_peak_) and peak work rate (WR_peak_) were 11.9 ± 4.0 ml/kg/min (45 % of predicted V'O_2max_), 117 ± 32 beats/min (72 % of predicted HR_max_) and 22.5 ± 13.0 W, respectively. Peak ratings of perceived exertion (RPE) were on the range "hard" to "very hard". All 8 patients reached their limit of functional capacity in terms of either their cardiopulmonary or neuromuscular performance.

A ventilatory threshold (VT) was identified in 7 patients and a respiratory compensation point (RCP) in 6 patients: mean V'O_2_ at VT and RCP was 8.9 and 10.7 ml/kg/min, respectively, which represent 75 % (VT) and 85 % (RCP) of mean V'O_2peak_. Incremental CPET provided sufficient information to satisfy the responsiveness criteria and identification of key outcomes in all 8 patients.

For CLTs, mean steady-state V'O_2_ was 6.9 ml/kg/min (49 % of V'O_2_ reserve), mean HR was 90 beats/min (56 % of HR_max_), RPEs were > 2, and all patients maintained the active work rate for 10 min: these values meet recommended intensity levels for bouts of training.

**Conclusions:**

The augmented RATT is deemed feasible for incremental cardiopulmonary exercise testing and exercise training in dependent-ambulatory stroke patients: the approach was found to be technically implementable, acceptable to the patients, and it showed substantial cardiopulmonary responsiveness. This work has clinical implications for patients with severe disability who otherwise are not able to be tested.

## Background

Cardiopulmonary fitness is compromised in stroke patients: their peak oxygen uptake (V'O_2peak_) ranges from 8–22 mL/kg/min, which corresponds to approximately half of age and gender matched healthy controls [1, 2]. The low V'O_2peak_ limits patients' ability to live independently [3] and hinders participation in rehabilitation and exercise programmes [4]. Low cardiopulmonary fitness can further heighten the existing risk for cardiovascular disease [5] by predisposing patients to a sedentary lifestyle because of activity limitation and early fatigue [6].

A recent joint statement from the American Heart Association and the American Stroke Association recommends that stroke patients should undergo cardiopulmonary exercise testing (CPET) [4]. CPET delivers objective measures which allow accurate quantification of cardiorespiratory fitness, delineation of the physiological systems underlying exercise responses, and identification of exercise-limiting pathophysiological mechanisms [7]. CPET outcomes can also be used to evaluate the effects of a longitudinal training programme and to determine the training intensity for individualized exercise prescription [4, 8, 9]. However, impairments following stroke such as weakness, ataxia or spasticity can preclude some patients from exercise testing on standard devices. Semi-recumbent cycle ergometers and total-body recumbent steppers have hitherto been used as alternatives to standard treadmills and cycle ergometers in order to test patients with balance and coordination problems [4].

Despite increasing availability of adapted devices, suitable methods and data for patients who are severely disabled are lacking. This problem is clearly demonstrated in the systematic review by Smith et al. [1], where only 2 of the 42 studies included reported data from dependent-ambulatory patients. The authors pointed out that the exclusion of the severely disabled group may result in overestimation of cardiopulmonary fitness for the entire stroke population [1]. Another systematic review on the effects of cardiovascular exercise early after stroke pointed out that concepts to influence and evaluate cardiopulmonary fitness in severely disabled patients are still lacking [10]. These open questions are addressed in the present work.

A robotics-assisted tilt table (RATT) is a device used clinically for early rehabilitation in severely impaired and bedridden neurological patients. It tilts the patient upright, provides support with a body harness, promotes weight bearing on the feet and moves the legs in a cyclic stepping movement. To promote active participation during the rehabilitation process, we have augmented a RATT system to allow patients to see their exercise work rate together with a target work rate [11]. This approach was shown to be feasible for exercise testing both in normal subjects and in spinal cord injured patients [12, 13]. We hypothesized that the augmented RATT should enable stroke patients with severe motor weakness to be tested.

Incremental CPET aims to approach a person's limit of functional capacity in regard to cardiopulmonary and/or neuromuscular exertion. The main parameters investigated here, which can be determined from incremental CPET, include both peak and submaximal values:

- Peak: peak oxygen uptake (V'O_2peak_), which represents aerobic capacity; peak heart rate (HR_peak_); and peak work rate (WR_peak_), which is the highest volitional effort. Additional criteria are applied to V'O_2_ and WR responses to determine whether the observed peaks represent true maximal values.
- Submaximal: the 1^st^ ventilatory threshold, denoted here as VT, which provides an approximation of endurance capacity [7, 14]; and the 2^nd^ ventilatory threshold, denoted here as the respiratory compensation point (RCP), that occurs at the onset of hyperventilation [15].
- Subjective measures such as rating of perceived exertion (RPE) may also be recorded at intervals throughout the test.

This is the first study where the novel augmented RATT system was applied to severely-disabled patients. Therefore we wanted to investigate whether, with this new exercise testing modality, the principal incremental CPET outcomes can be identified and whether, during constant-load CPET, sustained exercise intensity meets recommendations for training.

The aim of this study was therefore to evaluate the feasibility of the augmented RATT for incremental cardiopulmonary exercise testing and exercise training in dependent-ambulatory stroke patients, i.e. those with severe physical disability who are unable to use standard devices. Criteria for the feasibility assessment were: (i) implementation – technical feasibility of the augmented RATT for exercise testing, (ii) acceptability – was the exercise tolerable?, and (iii) responsiveness – was there a measurable, high-level cardiopulmonary reaction?.

## Materials and methods

### Study design and participants

This descriptive, cross-sectional feasibility study was conducted at the Reha Rheinfelden, a rehabilitation centre in the north-west of Switzerland, from October 2013 to April 2014. The Ethics Review Committee of Canton Aargau, Switzerland, approved the study. All subjects gave their written informed consent before participating in the study.

Eight patients (4 female) aged 58.3 ± 9.2 years (mean ± SD) were recruited and all completed the study. The mean Functional Ambulation Category (FAC, [16]) was 1.8 (range 0–3; Table 1). Patient inclusion criteria were: (1) a diagnosis of first-ever stroke, either ischaemic or intracerebral haemorrhage by radiologic evidence; (2) > 2 months post stroke; (3) > 18 years old; (4) dependent in ambulation with Functional Ambulation Category (FAC) ≤ 3; (5) Mini Mental State Examination (MMSE, [17]) score > 20 (cognitive function); and (6) willing to cooperate in the study and able to attend all testing sessions. Exclusion criteria were: (1) any contraindications to maximal exercise testing according to the American College of Sports Medicine guidelines [18]; (2) any contraindications for the RATT based on guidelines from the manufacturer; (3) severe aphasia or other communication problems; and (4) severe concurrent pulmonary disease. A cardiologist reviewed all prospective subjects for cardiac status before giving approval for formal enrolment.

**Table 1:** Characteristics and demographic data of subjects (*n* = 8)

Characteristic	Value
Age (years)	58.3 ± 9.2
Sex, *n* (%)	
Male	4 (50 %)
Female	4 (50 %)
Height (cm)	167.6 ± 8.8
Body mass (kg)	75.2 ± 7.4
Body mass index (kg/m^2^)	26.9 ± 3.3
Type of stroke, *n* (%)	
Ischaemic	5 (62.5 %)
Haemorrhagic	3 (37.5 %)
Hemiparetic side, *n* (%)	
Left	4 (50 %)
Right	3 (37.5 %)
Bilateral	1 (12.5 %)
Years post stroke, median (IQR)	1 y 42 d (8.2 y)
FAC, mean (range)	1.8 (0–3)
MMSE score	27.1 ± 3.2
Comorbidities, *n* (%)	
Diabetes mellitus	1 (12.5 %)
Hypertension	5 (62.5 %)
Dyslipidemia	2 (25 %)
None	3 (37.5 %)
Antihypertensive medications, *n* (%)	
β-blocker	1 (12.5 %)
ACE inhibitors	3 (37.5 %)
Calcium channel blockers	1 (12.5 %)
None	3 (37.5 %)

Values are mean ± SD unless otherwise indicated

Abbreviations: n, number; SD, standard deviation; MMSE, Mini Mental State Examination; IQR, Interquartile range; FAC, Functional Ambulation Category; ACE, angiotensin-converting-enzyme

### Robotics-assisted tilt table (RATT)

A RATT system (Erigo, Hocoma AG, Switzerland) was augmented to facilitate active participation during exercise. The basic RATT is a motorized tilt table with a body harness to support the body and two motor drives to support cyclical movement of the legs. Two thigh cuffs fix the legs and interface to the leg drives, and two foot plates support the feet. The RATT is designed to be used for early rehabilitation in neurological patients to provide early mobilization and intensive sensorimotor stimulation. Additionally, it is also claimed to enhance cardiovascular output by cyclic leg loading. During the therapy, the RATT can be tilted up from 0 to 80° and the cyclic leg movement can be set to a stepping cadence between 8 and 80 steps/min.

**Fig. 1:**
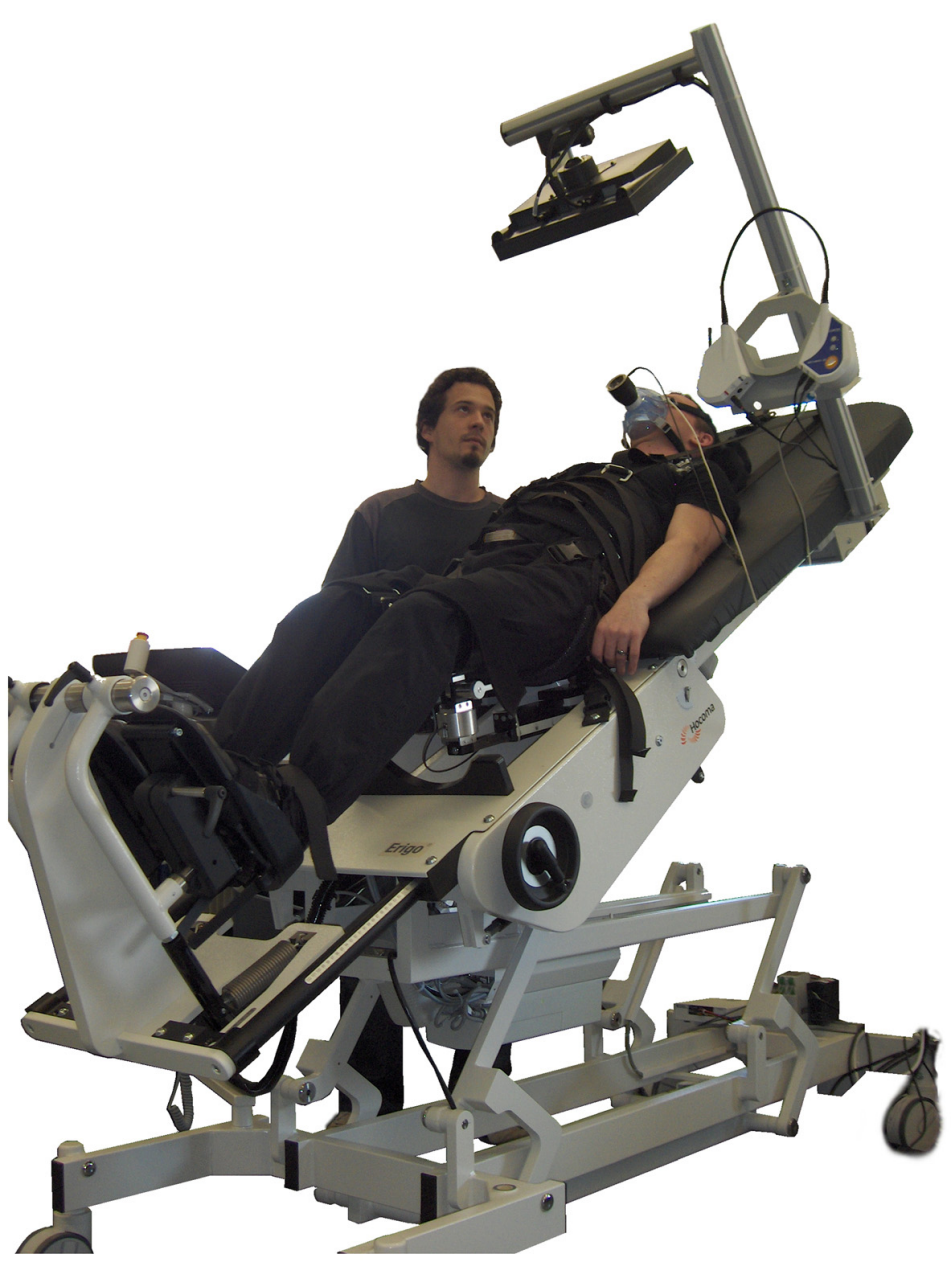
A modified robotics‐assisted tilt table (RATT) with force sensors under the thigh cuffs, visual feedback system and breath‐by‐breath cardiopulmonary monitoring system

With the augmented RATT system, patients are able to see their exercise work rate together with a target work rate [11]. This was achieved by adding individual force sensors to the left and right leg cuffs, a work rate estimation algorithm and a real time visual feedback system (Fig. 1). Patients were instructed to adapt their volitional leg effort to follow the target. Active exercise is achieved by producing forces into the leg cuffs in synchrony with the movement of the RATT.

### Experimental procedures

Patients took part in three exercise sessions, each separated by a minimum of 24 h: a familiarization, an incremental exercise test (IET), and a constant load test (CLT). Patients were instructed to avoid strenuous activity within the 24 h before the test sessions and not to consume a large meal, caffeine or nicotine in the three hours prior to testing [19].

For each test, the patient was first transferred and secured to the RATT in accordance with the manufacturer's guidelines. Then the additional measurement systems (i.e. automatic blood pressure monitoring, and mask for breath-by-breath gas analysis) were fitted. The patient was then tilted upwards to 60°. During the test, the stepping cadence was set at 80 steps/min.

The familiarization was to instruct patients regarding the RATT, the measurement systems and the test procedures. It included a short ramp phase of 5-min duration and work rate ramp of 3 W/min to allow estimation of an appropriate ramp rate for the subsequent IET.

The IET consisted of: (1) a recorded rest phase, where the patient lay passively on the RATT for 3 min; (2) a passive phase, where the RATT moved the patient's legs for 5 min; (3) a ramp phase, where the patients actively moved their legs in synchrony with the RATT motion while attempting to follow the linearly increasing work rate target. The work rate ramp was set individually in the range of 1.2 to 3.5 W/min based on observations from the ramp phase of the familiarization with the aim to bring the patient to their functional limit within 8–12 min; and (4) a recovery phase, similar to the initial passive phase, where the RATT moved the patient's legs for 5 min. The termination criteria for the ramp phase followed the American College of Sports Medicine guidelines [18]. Additionally, blood pressure (BP) was used as a termination criterion: systolic BP > 210 mmHg or diastolic BP > 115 mmHg [20].

The CLT consisted of: (1) a rest phase for 3 min; (2) a passive phase for 5 min; (3) a constant load phase, where the patient actively moved their legs in synchrony with the RATT motion to follow the constant target work rate (the work rate was set at 40 % of peak work rate (WR_peak_) obtained from the IET) for 10 min; and (4) a recovery phase for 5 min.

### Outcome measures

Metabolic gas exchange was recorded using a breath-by-breath system (MetaMax 3B, Cortex Biophysik GmbH, Germany) and outcome variables were analysed using the associated Metasoft software. Prior to each test, pressure, volume and gas calibration were performed: volume was calibrated using a 3-L syringe and gas was calibrated using ambient air and a certified precision gas mixture (15 % oxygen and 5 % carbon dioxide) according to the manufacturer's instructions. Heart rate was continuously recorded using a chest strap (model T34, Polar Electro Oy, Finland). Blood pressure was measured by automatic sphygmomanometry (HEM 907XL, Omron Corporation, USA) every 2 min during the tests.

Outcome measures for IETs were as follows. Peak oxygen uptake (V'O_2peak_) was determined as the maximum of a 30-s average during the ramp phase. The peak respiratory exchange ratio (RER_peak_) was the 30-s average of RER at the time of V'O_2peak_. Peak heart rate (HR_peak_) was defined as the highest value of HR reached during the ramp phase. The peak work rate (WR_peak_) was calculated as the maximum of a 10-s moving average of the recorded work rate.

The first and second ventilatory thresholds (VT and RCP) were determined independently by two experienced raters (JSa and KH) using the methods described by Binder et al. [21] and summarised in the following paragraphs, and the average of the two ratings was taken. The thresholds were characterized by the value of V'O_2_ at the point where the criteria given below were deemed to be fulfilled. Threshold analysis was carried out visually based on 15-breath averages of the variables concerned.

The first ventilatory threshold, i.e. that which is denoted here as the VT, was determined graphically using the combination of these criteria: (1) the point at which the ventilatory equivalent for oxygen (V'E/V'O_2_) reaches a minimum or has its first increase without a simultaneous increase in the ventilatory equivalent for carbon dioxide (V'E/V'CO_2_); (2) the point at which partial pressure of end-tidal oxygen tension (P_ET_O_2_) reaches a minimum or has its first increase without a decrease in the partial pressure of end-tidal carbon dioxide tension (P_ET_CO_2_); and, (3) the deflection point of carbon dioxide output (V'CO_2_) versus oxygen uptake (V'O_2_; V-slope method). When these 3 criteria gave different results, the first two were prioritized.

The second ventilatory threshold, i.e. the respiratory compensation point (RCP), was determined graphically by inspection of: (1) the minimal value or nonlinear increase of V'E/V'CO_2_; (2) the turning point of P_ET_CO_2_; and, (3) the point of deflection of minute ventilation (V'E) versus V'CO_2_ [21]. Again, if these 3 criteria gave different results, the first two were prioritized.

Borg CR10 ratings of perceived exertion (RPE) for dyspnea and leg effort [22] were recorded every 3 min during the tests. The reasons for test termination were recorded.

Outcome measures for CLTs were the steady-state V'O_2_ and heart rate during the rest, passive and constant load phases. The accuracy of the patient's achievement of target work rate was quantified by the root mean square error (RMSE) between the target and actual work rates between the first and ninth min of the constant load phase. The Borg CR10 RPE ratings for dyspnea and leg effort were recorded.

### Criteria to determine feasibility of the RATT for exercise testing and training

The criteria for feasibility assessment were [23]: (i) implementation (technical feasibility of the augmented RATT for exercise testing), (ii) acceptability (was the exercise tolerable?), and (iii) responsiveness (was there a measurable, high-level cardiopulmonary reaction?).

Incremental CPET was considered to have satisfied responsiveness criteria if in a given patient at least one of the following outcomes could be identified from the IETs (adapted from Marzolini et al. [8]): V'O_2max_, WR_max_, VT or RCP.

The first two of these, V'O_2max_ and WR_max_, indicate whether a patient's functional capacity in terms of cardiopulmonary and/or neuromuscular exertion was reached:

- V'O_2max_ was deemed to have been achieved if at least one of the following was observed: plateau in V'O_2_ (increase in V'O_2_ less than 150 mL in the final minute of exercise [24]), RER ≥ 1.10 (or RER ≥ 1.05 for age ≥ 50 [25]) or HR_peak_ ≥ HR_max_ – 10 [8]. Here, HR_max_ was obtained from an age-related prediction formula [18].
- Achievement of WR_max_ was marked by a plateau in WR with the patient no longer able to reach the WR target.

Constant-load CPET was considered to have satisfied responsiveness criteria if the intensity and duration of steady-state exercise during CLTs was found to have met current recommendations for exercise and physical activity after stroke; these are defined as 40 to 70 % of V'O_2_ reserve or HR reserve; or, 55 to 80 % of HR_max_; or, RPE of 11 to 14 on the Borg scale (6–20) [4]. V'O_2_ reserve is defined as V'O_2peak_ – V'O_2rest_ and HR reserve as HR_peak_ – HR_rest_ [18].

### Statistical analysis

Descriptive statistics were used to evaluate the distribution of the variables. Continuous variables are presented as mean ± standard deviation. Categorical variables are presented as frequencies and percentages. All analyses were performed using SPSS version 19 (IBM Corporation, USA).

## Results

(i) Implementation: The augmented RATT could be successfully used to implement both the IET and the CLT in stroke patients without the need to further modify the system. There were no technical problems that interrupted the tests.
(ii) Acceptability: The patients could understand the task to keep up with the work rate target using the visual feedback system and adaptation of their volitional leg effort. All patients could exercise until the end of the protocols without any complications. All tests were completed successfully according to the termination criteria (IET) or pre-specified duration (CLT).
(iii) Responsiveness: IET (*n* = 8; Table 2): Absolute V'O_2peak_ was 845 ± 266 mL/min (relative V'O_2peak_ was 11.9 ± 4.0 mL/kg/min), which corresponds to 45.2 % of the expected V'O_2max_ based on the prediction method of Wasserman et al. [14]. HR_peak_ was 117 ± 32 beats/min, which is 72.0 % of the predicted value (Table 2). WR_peak_ was 22.5 ± 13.0 W. The average ratings of perceived exertion (RPE, Borg CR10) for dyspnea and leg effort at the end of the ramp phase were 5.4 and 6.6, respectively; these lie on the qualitative range of "hard" to "very hard".

**Table 2:** Summary of outcome variables from incremental exercise tests (*n* = 8)

Outcome variable	Value	Range
Peak exercise variables		
V'O_2peak_ absolute (mL/min)	844.8 ± 265.7	352.0 – 1176.0
V'O_2peak_ relative (mL/min/kg)	11.9 ± 4.0	5.6 – 17.5
V'O_2peak_ as % of predicted V'O_2max_ [14]	45.2 ± 8.8	32.3 – 58.2
HR_peak_ (beats/min)	117.3 ± 31.5	66.0 – 155.0
HR_peak_ as % of predicted HR_peak_ (220-age)	72.0 ± 17.7	44.9 – 99.3
SBP (mmHg)	192.9 ± 29.9	140.0 – 220.0
DBP (mmHg)	88.5 ± 12.7	78.0 – 110.0
Rate-pressure product	230.0 ± 79.8	92.4 – 341.0
RER_peak_	1.00 ± 0.10	0.78 – 1.20
Borg CR10 RPE scale dyspnea	5.4 ± 2.8	1.0 – 9.0
Borg CR10 RPE scale leg effort	6.6 ± 1.9	4.0 – 9.0
Oxygen cost of work (mL/min/W) (*n* = 6)	21.9 ± 3.8	16.0 – 26.0
WR_peak_ (W)	22.5 ± 13.0	5.5 – 38.6
Time to V'O_2peak_ (sec)	742.5 ± 161.8	600.0 – 1080.0
Submaximal exercise variables (*n* = 7)		
Absolute V'O_2_ at VT (mL/min)	677.1 ± 187.1	520.0 – 1053.0
Relative V'O_2_ at VT (mL/kg/min)	8.9 ± 2.9	6.9 – 15.0
VT as % of V'O_2peak_	75.1 ± 17.6	52.3 – 100.8
RER at VT	0.88 ± 0.08	0.78 – 0.96
Absolute V'O_2_ at RCP (mL/min) (*n* = 6)	800.3 ± 206.7	547.0 – 1136.0
Relative V'O_2_ at RCP (mL/kg/min) (*n* = 6)	10.7 ± 3.1	7.3 – 16.2
RCP as % of V'O_2peak_ (*n* = 6)	84.8 ± 11.6	70.6 – 99.3
RER at RCP (*n* = 6)	1.02 ± 0.13	0.84 – 1.24

Values are mean ± SD

Abbreviations: V'O_2peak_, peak oxygen uptake; V'O_2_, oxygen uptake; HR, heart rate; HR_peak_, peak heart rate; RER_peak_, peak respiratory exchange ratio; SBP, systolic blood pressure; DBP, diastolic blood pressure; RPE, rating of perceived exertion; WR_peak_, peak work rate; VT, 1^st^ ventilatory threshold; RER, respiratory exchange ratio; RCP, respiratory compensation point

Reasons for termination of the IET were: leg fatigue (*n* = 4, 50 %); abnormal blood pressure, i.e. systolic BP > 210 mmHg, (*n* = 2, 25 %); breathing effort (*n* = 1, 12.5 %); and generalized fatigue (*n* = 1, 12.5 %).

The VT was identified in 7 patients (4 female) and the RCP in 6 patients (3 female). The average absolute V'O_2_ values at VT and RCP were 677 and 800 mL/min (relative V'O_2_ at VT and RCP was 8.9 and 10.7 mL/kg/min) which represent 75.1 % (VT) and 84.8 % (RCP) of mean V'O_2peak_.

Incremental CPET provided sufficient information to satisfy the responsiveness criteria, i.e. V'O_2max_, WR_max_, VT or RCP were able to be determined, in all 8 patients (4 female, 4 male): all 8 patients reached the limit of functional capacity in terms of either V'O_2max_ (7 patients – 4 female) or WR_max_ (6 patients – 3 female). Of the 7 patients deemed to have satisfied the criteria for V'O_2max_, 5 reached a plateau, 3 met the RER criterion and 1 met the HR_max_ criterion.

**Fig. 2:**
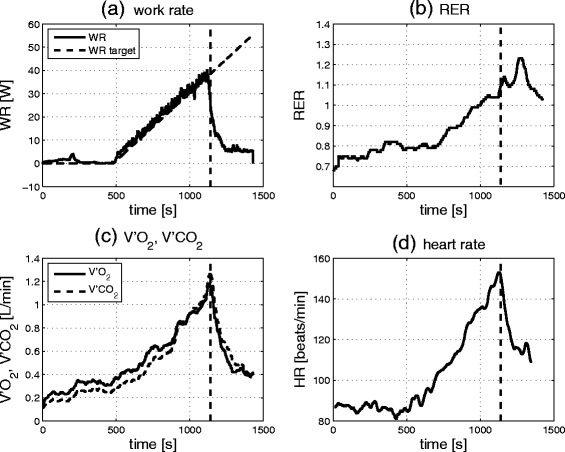
Typical peak cardiopulmonary responses (Subject 8) during the IET test protocol. **a** Target and measured work rates, **b** respiratory exchange ratio (RER), **c** oxygen uptake (V'O_2_) and carbon dioxide output (V'CO_2_), **d** heart rate (HR). The plots of RER, V'O_2_, V'CO_2_ and HR show averages over a 30s moving window

**Fig. 3:**
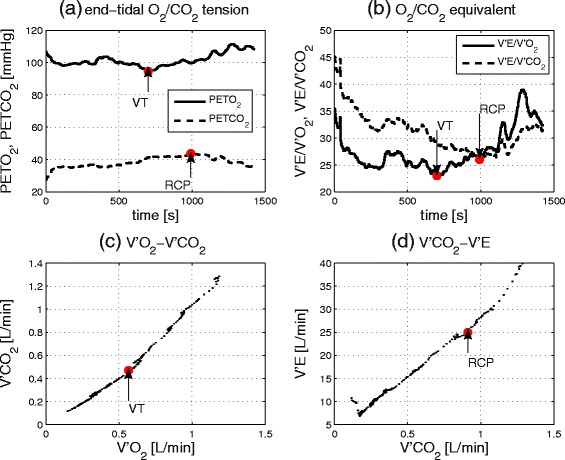
Determination of the 1^st^ ventilatory threshold (VT) and the respiratory compensation point (RCP) from Subject 8. **a** VT is at the minimal value of P_ET_O_2_ and RCP at the turning point of P_ET_CO_2_, **b** VT is at the minimal value of V'E/V'O_2_ and RCP at the minimal value of V'E/V'CO_2_, **c** VT is at the deflection point of V'CO_2_ vs. V'O_2_ ('V-slope method'), **d** RCP is at the deflection point of V'E vs. V'CO_2_

To illustrate typical IET responses, Fig. 2 shows the target and measured work rates as well as cardiopulmonary responses from the IET in Patient 8*.* Fig. 3 shows the graphical plots for determination of the VT and the RCP in the same patient.

CLT (*n* = 8; Table 3): The transition from passive to constant load exercise yielded a higher increase in V'O_2_ (2.7 mL/kg/min) than did the transition from rest to passive (0.9 mL/kg/min) (Table 3). During the active phase of the exercise, all patients were able to achieve the recommended training intensity level [4] based on percentage of V'O_2_ reserve, percentage of HR_max_, and RPE: the constant work rate was set at 40 % of individual WR_peak_ values which resulted, on average, in a steady-state V'O_2_ of 49 % of V'O_2_ reserve, steady-state HR of 56 % of predicted HR_max_ and RPE > 2. All patients could maintain the active work rate for 10 min as prescribed. The accuracy of maintaining the work rate target (RMSE) was 1.3 W.

**Table 3:** Summary of outcome variables from constant load tests (*n* = 8)

Outcome variables	Values
Initial rest phase	
V'O_2_ absolute (mL/min)	243.9 ± 33.6
V'O_2_ relative (mL/min/kg)	3.30 ± 0.43
HR (beats/min)	73.8 ± 9.8
Passive phase	
V'O_2_ absolute (mL/min)	316.6 ± 75.9
V'O_2_ relative (mL/min/kg)	4.20 ± 0.78
HR (beats/min)	76.3 ± 10.0
Constant load phase	
V'O_2_ absolute (mL/min)	519.5 ± 117.7
V'O_2_ relative (mL/min/kg)	6.9 ± 1.6
V'O_2_ as % of V'O_2_ reserve	48.7 ± 19.1
HR (beats/min)	90.3 ± 18.6
HR as % of HR reserve	34.8 ± 32.0
HR as % of HR_max_	56.0 ± 9.9
WR (W)	11.5 ± 6.1
Borg CR10 RPE scale dyspnea	2.1 ± 1.0
Borg CR10 RPE scale leg effort	2.9 ± 0.6
RMSE of WR (W)	1.3 ± 1.0

Values are mean ± SD

Abbreviations: V'O_2_, oxygen uptake; HR, heart rate; WR, work rate; W, Watts; RPE, rating of perceived exertion; RMSE, root mean square error

## Discussion

The aim of this study was to evaluate the feasibility of the augmented RATT for incremental cardiopulmonary exercise testing and exercise training in dependent-ambulatory stroke patients. Feasibility assessment considered technical feasibility, patient tolerability, and cardiopulmonary responsiveness.

### Feasibility for incremental cardiopulmonary exercise testing

For all 8 patients tested, incremental CPET provided sufficient information to satisfy the responsiveness criteria, i.e. V'O_2max_, WR_max_, VT or RCP were successfully identified. All 8 patients also reached their limit of functional capacity due to either cardiopulmonary limitations (V'O_2max_ criteria; 7 patients – 4 female) or neuromuscular limitations (WR_max_ criteria; 6 patients – 3 female). Of these 8 patients, 5 reached both sets of criteria for cardiopulmonary and neuromuscular capacity, 2 patients satisfied only the cardiopulmonary criteria, and 1 patient reached only the neuromuscular limitation.

It is interesting that, in these numbers, female patients are at least as highly represented as males. Marzolini et al. [8] previously noted that females after stroke were much less likely than males to achieve similar feasibility criteria from baseline CPETs: 40 % for females vs. 81 % for males. That difference was attributed to greater disability and weakness in the females examined in a study of mildly-impaired patients. The results herein, with severely-impaired patients, indicate that such measurement difficulties can be overcome by employing appropriate testing equipment, i.e. the augmented RATT. However, more subjects are required to reliably study these male–female ratios in the outcomes.

The V'O_2peak_ reported in this study (11.9 ± 4.0 mL/kg/min) is lower than values previously reported in ambulatory stroke patients using: cycle ergometry, 17.2 ± 3.0 mL/kg/min [26]; recumbent cycle ergometry, 16.0 ± 1.2 mL/kg/min [27]; or a treadmill with body weight support, 14.4 ± 5.1 mL/kg/min [24]. This low value may be attributable in part to the more profound disability in the patients in the present study and in part to the observation that, in normal subjects, the RATT V'O_2peak_ is approximately 20 % lower than with a cycle ergometer and 30 % lower than for a treadmill [28].

HR_peak_ was on average 72 % of the predicted value. The rate-pressure product, which reflects the cardiovascular load during exercise, was 230. These results are comparable to previously documented results in mildly to moderately disabled stroke patients [2, 24, 26, 29]. This suggests a similar myocardial work load.

Successful identification of a VT (7 patients – 4 female) and/or an RCP (6 patients – 3 female) from incremental CPET provides an additional means of prescribing exercise intensity. The relative V'O_2peak_ at the VT (8.9 mL/kg/min) is lower than previous reports [8, 20], which may be due to the same reasons as described above in relation to lower V'O_2peak_. The VT as a percentage of V'O_2peak_ found in this study is in line with other studies which reported values in the range 73.4 to 89.7 % of V'O_2peak_ [8, 20, 30].

### Feasibility for exercise training

Current standards for prescription of exercise intensity for stroke patients are derived from a subset of the main incremental CPET outcomes. For stroke patients, these are [4]: 40 to 70 % of V'O_2_ reserve or HR reserve; or, 55 to 80 % of maximal HR; or, RPE between 11 and 14 (6 to 20 scale), which corresponds approximately to 2 to 4.5 on the Borg CR10 RPE scale. It is recommended that this intensity level should be reached on 3 to 5 days per week using 20 to 60 min per exercise session or by multiple 10-min sessions.

The CLTs demonstrate that, during the active phase of the exercise, all patients were able to sustain the recommended intensity level based on percentage of V'O_2_ reserve, percentage of HR_max_, and RPE for 10 min: the constant work rate was set at 40 % of individual WR_peak_ values which resulted, on average, in a steady-state V'O_2_ of 49 % of V'O_2_ reserve, steady-state HR of 56 % of HR_max_ and RPE > 2.

During the passive phases of the CLTs the exercise intensity was far below the recommended levels: passive V'O_2_ was on average 18 % of V'O_2_ reserve and passive HR was 9 % of HR reserve. This demonstrates that muscle activation is very low during passive movement and emphasises the need for active participation of the patient using the work rate biofeedback screen implemented within the augmented RATT. This low intensity of passive motion confirms a previous report with robotics-assisted treadmill exercise [31]. With this low intensity, patients cannot effectively improve their cardiopulmonary fitness.

These considerations show that the augmented RATT is a feasible platform for implementation of a prescribed exercise training programme where 10-min bouts of exercise form part of the recommendations, but future work is required to investigate the response to a longitudinal training intervention using the RATT.

### Limitations

The RATT V'O_2peak_ was previously observed to be approximately 20 % lower than the cycle ergometer and 30 % lower than the treadmill in normal subjects [28], but it is not certain whether these differences would be the same in stroke patients. Further study is needed to address the comparability of the RATT and the standard exercise testing devices (e.g. cycle ergometer) in patients who are capable of using conventional modalities.

It was observed here that the efficiency of work production on the RATT, as characterized by the inverse of the oxygen cost of the work (mean value 21.9 mL/min/W, Table 2), is substantially lower than for cycle or treadmill ergometry. This was also observed previously in able-bodied subjects on the RATT [12]. This is probably due to a combination of factors including the way the muscle groups are activated and the employment of a possibly non-optimal exercise cadence. Here, a cadence of 80 steps/min was used as this is the highest rate allowed by the device employed. To improve efficiency, a higher stepping rate might be desirable, so that lower forces are needed for a given work rate target (because work rate is the product of torque and angular velocity).

The small sample size in this study may limit generalizability of the data describing cardiopulmonary fitness in dependent-ambulatory stroke patients. The V'O_2peak_ in this study may still be an overestimate in relation to the overall population because we did not include patients who had heart disease or who were not approved by the cardiologist for cardiac safety. Additionally, the mean age of the patients (58.3 years) was slightly lower than the general stroke population reported in high income countries (66 years) [32] or in Switzerland (72.5 years) [33].

## Conclusion

The augmented RATT is deemed feasible for incremental cardiopulmonary exercise testing and exercise training in dependent-ambulatory stroke patients: the approach was found to be technically implementable, it was well tolerated by the patients (acceptability), and substantial cardiopulmonary responses were observed (responsiveness).

## Abbreviations

CPET: Cardiopulmonary exercise testing
CLT: Constant load test
HR: Heart rate
HR_peak_: Peak heart rate
HR_max_: Maximal heart rate
IET: Incremental exercise test
P_ET_CO_2_: Partial pressure of end-tidal carbon dioxide tension
P_ET_O_2_: Partial pressure of end-tidal oxygen tension
RATT: Robotics-assisted tilt table
RCP: Respiratory compensation point
RER_peak_: Peak respiratory exchange ratio
RMSE: Root mean square error
RPE: Rating of perceived exertion
V'CO_2_: Carbon dioxide output
V'E: Minute ventilation
V'O_2_: Oxygen uptake
V'O_2max_: Maximal oxygen uptake
V'O_2peak_: Peak oxygen uptake
VT: Ventilatory threshold
WR_peak_: Peak work rate
